# Synthesis of a novel glucose capped gold nanoparticle as a better theranostic candidate

**DOI:** 10.1371/journal.pone.0178202

**Published:** 2017-06-05

**Authors:** Saritha Suvarna, Ujjal Das, Sunil KC, Snehasis Mishra, Mathummal Sudarshan, Krishna Das Saha, Sanjit Dey, Anindita Chakraborty, Y. Narayana

**Affiliations:** 1 Department of Studies in Physics, Mangalore University, Mangalagangotri, Karnataka, India; 2 Department of Physiology, Centre for Nanoscience and Nanotechnology and Centre with Potential for Excellence in Particular Area (CPEPA), University of Calcutta, Kolkata, West Bengal, India; 3 Cancer Biology & Inflammatory Disorder Division, IICB, Kolkata, West Bengal, India; 4 UGC-DAE Consortium for Scientific Research Centre, Kolkata, West Bengal, India; Institute of Materials Science, GERMANY

## Abstract

Gold nanoparticles are predominantly used in diagnostics, therapeutics and biomedical applications. The present study has been designed to synthesize differently capped gold nanoparticles (AuNps) by a simple, one-step, room temperature procedure and to evaluate the potential of these AuNps for biomedical applications. The AuNps are capped with glucose, 2-deoxy-D-glucose (2DG) and citrate using different reducing agents. This is the first report of synthesis of 2DG-AuNp by the simple room temperature method. The synthesized gold nanoparticles are characterized with UV-Visible Spectroscopy, Fourier transform infrared spectroscopy (FTIR), Transmission electron microscopy (TEM) and selected area electron diffraction (SAED), Dynamic light scattering (DLS), and Energy-dispersive X-ray spectroscopy (SEM-EDS). Surface-enhanced Raman scattering (SERS) study of the synthesized AuNps shows increase in Raman signals up to 50 times using 2DG. 3-(4, 5-dimethylthiozol-2-yl)-2,5-diphenyl tetrazolium bromide (MTT) assay has been performed using all the three differently capped AuNps in different cell lines to assess cytotoxcity if any, of the nanoparticles. The study shows that 2DG-AuNps is a better candidate for theranostic application.

## Introduction

Application of nanotechnology has gained a major thrust in research especially in the area of medicine and biology. With several gold nanoparticle based therapies currently undergoing clinical trials, gold nanoparticles have become the subject of a wide ranging international research effort with preclinical studies underway [1]. Synthesis of nanoscale structures of inert metals like gold are of great interest for the present day researchers as gold possess certain physical properties which are suitable for several biomedical applications. Thus gold nanoparticles show significant future promise in the fields of diagnostic imaging and therapy, including multifunctional drug-delivery vehicles [2–8], The Au-ZnO nanocomposite exhibits significant enhancement in the Raman signals for C_70_ C_70_ molecules [9]. Various standard protocols and recent advances for shape-controlled synthesis of nanocrystals are reported, it is clear that significant progress has been made towards design synthesis of nanocrystals, with desired shape, crystallity and composition by controlling the nucleation and growth process using specific synthetic protocols[10]. Studies also reported doses (concentration) of Au-Nps required for controlling the growth or decay of C_2_C_12_ myoblast cells. The obtained results clearly demonstrate that the treatments with nanoparticles diminished the growth of cells. Apoptosis was determined to be enhanced with an increase in nanoparticle concentration, and a significant concentration of nanoparticles resulted in cellular death [11]. Additionally, the galvanic replacement reaction with HAuCl_4_ in an organic medium was implemented to prepare hydrophobic hollow Au-Ag nanocages with tunable localized surface Plasmon resonances [12]. The superiority of atom beam sputtering over in beam mixing and ion implantation in the synthesis of Au nanoparticles has also been demonstrated. The possibility of using Au-SiO_2_ nanocomposites as a biosensor for the detection of ovarian cancer cells has been explored [13]. Studies also lay special emphasis on the uniqueness of the gold nanoparticles for treatment of life threatening diseases like cancer [14]. Available reports show potential of gold nanoparticles in the field of photodynamic therapy due to their ability of producing heat to kill the tumors [15]. AuNPs are reported as good photo-thermal agents for cancer therapy because they show efficient local heating upon excitation of surface plasmon oscillations. The strong absorption, efficient heat conversion, high stability, inherent low toxicity and well-defined surface chemistry of AuNps, contribute to the growing interest in their photothermal therapy (PTT) applications [16]. In the recent past, researchers also discussed the near-infrared-active AuNps, which include different shape of nanoparticle systems, especially regarding the clinical translation of AuNp[17]. Varity of shapes have driven a new wave of interest in their optical properties and thus offers applications like imaging and spectroscopic detection of cancer [18,19] Urchin-shaped gold nanoparticles deserves special mention as this type of the AuNps may find applications as materials for Surface Enhanced Raman Scattering (SERS)[20]. The gold supraparticles have nanoparticle building blocks in close contact that generate highly intense SERS signals. They also generate plasmonic heat more efficiently and kill more cancer cells than the constituent nanoparticle building blocks. These traits make the proposed crystallographically aligned supraparticles promising candidates for nanomedicine applications such as SERS -based diagnostics and plasmonics-based theranostics [21]. Studies also reported generalized application of gold nanostars for ultrasensitive identification of molecules based on both localized surface Plasmon resonance (LSPR) and surface enhanced Raman scattering (SERS) are the requirements of plasmonic sensors, related to sufficiently large areas where nanoparticles are uniformly immobilized with high density, as well as mechanical flexibility, which offers additional advantages for real world applications [22]. However, although gold is biologically inert and thus shows much less toxicity as compared to other metal nanoparticles, gold has a relatively lower rate of clearance from circulation and hence can pose serious deleterious effects on health [23]. Recent studies showed that AuNPs can cross the blood-brain barrier, interact with the DNA and even produce genotoxic effects [24]. As such, surface modifications of AuNps are gaining attention in current day research programs where attaching a ligand and/or capping of the AuNps could help making the particles more biocompatible so as to achieve specific targeting of diseased cells and tissues [25,26]. Synthesizing AuNps with lesser toxicity is now one of the primary interests in the field of nanotechnology for its applicability in biomedical sciences. Based on this perspective, the present work has been designed to formulate a synthesis of a novel glucose capped gold nanoparticle which can be considered as a better theranostic candidate.

The glucose analog 2-deoxy-D-glucose (2DG), an inhibitor of glycolytic ATP production and glucose transport is the most widely reported, metabolic inhibitor for targeting glucose metabolism. In addition, 2DG can induce oxidative stress [27], inhibits N-linked glycosylation and induces apoptosis via endoplasmic reticulum(ER) stress [28]. It can efficiently slow cell growth and potently facilitate apoptosis in specific cancer cells. Although 2DG itself has limited therapeutic effect in many types of cancers, it may be combined with other therapeutic agents or radiotherapy[29] to exhibit a synergistic anticancer effect [30]. Some earlier studies described the Warburg effect and discussed about 2DG and its underlying mechanisms as potential application for cancer treatment [31]. 2-deoxy-D-glucose labeled gold nanoparticles have been shown to provide high-resolution metabolic and anatomic information of tumor in a single CT scan [32,33]. The importance of computed tomography (CT) as one of the leading radiology techniques applied in the field of biomedical imaging escalated the development of nanoparticles as the next generation CT contrast agents [34–37]. Studies showed that glucose capped gold nanoparticles has been specially chosen to target cancer cells, as such capped nanoparticles show faster cellular uptake in cancer cells. Additionally, larger numbers of glucose molecules are internalized via glucose transporter (GLUT) receptors present on the cancer cell surface [38,39]. It is already established that brain tumor cells have over expression of the facilitative glucose transporter protein (GLUT) [40]. Recently 2-deoxy-D-glucose modified poly (ethylene glycol)-co-poly(trimethylene carbonate) nanoparticles (DGlu-Np) were developed as a potential dual-targeted drug delivery system for enhancing the blood brain barrier penetration via Glut-mediated transcytosis and improving the drug accumulation in the glioma via GLUT-mediated endocytosis [41]. Our present paper describes the synthesis of differently capped gold nanoparticles and characterization of the nanoparticles by different techniques. Idea of developing 2DG-capped AuNps is inspired by the classic citrate method and the greener glucose method for gold nanoparticle preparation. The primary motivation behind the work has been to develop a simple novel method of capping of AuNps and further to compare the citrate- and glucose-reducing gold nanoparticles with 2DG capped AuNps to determine the most potential candidate amongst these AuNps for biomedical applications. Evaluation of biocompatibility of the nanoparticles has been carried out using HeLa, HepG2 and HCT 116 cell lines.

## Materials and methods

### Materials

HAucl_4._3H_2_O (≥ 99.9%, Sigma Aldrich, USA), β-D-glucose (≥ 99.9%, Sigma Aldrich, USA), 2-deoxy-D-glucose (≥98% (GC), crystalline, Sigma Aldrich, USA)), Sodium Hydroxide (NaOH), (Merck Chemicals) are used in the present study. HeLa (Cervical Carcinoma), HCT 116 (Human Colorectal Carcinoma) and Hep G2 (Human liver Carcinoma) Cell lines are purchased from NCCS, Pune, India and cultured in DMEM medium supplemented with 10% fetal bovine serum (FBS) and 1% antibiotic (PSN) at 37°C in a humidified atmosphere under 5% CO_2_. After 75–80% confluency, cells, harvested with 0.025% trypsin and 0.52 mM EDTA in phosphate buffered saline(PBS), are seeded at required density to allow them to re-equilibrate a day before the start of experiment.

### Methodology

#### Synthesis of Au-Nps

Synthesis of Gu-AuNPs: Glucose capped AuNPs are synthesized by chemical route using HAuCl_4_ and β-D-Glucose as described by earlier researchers [42]. The aqueous solution of 0.05 M HAucl_4._3H_2_O is added to β-D-glucose (0.03 M) and stirred for 30 minutes. Subsequently, 0.5 M sodium hydroxide (NaoH) is added for completing reduction of gold salt. This resulted in a red colored solution of Glu-AuNps. β-D-glucose acted as reducing as well as capping agent in the AuNp synthesis.

**Fig 1:**
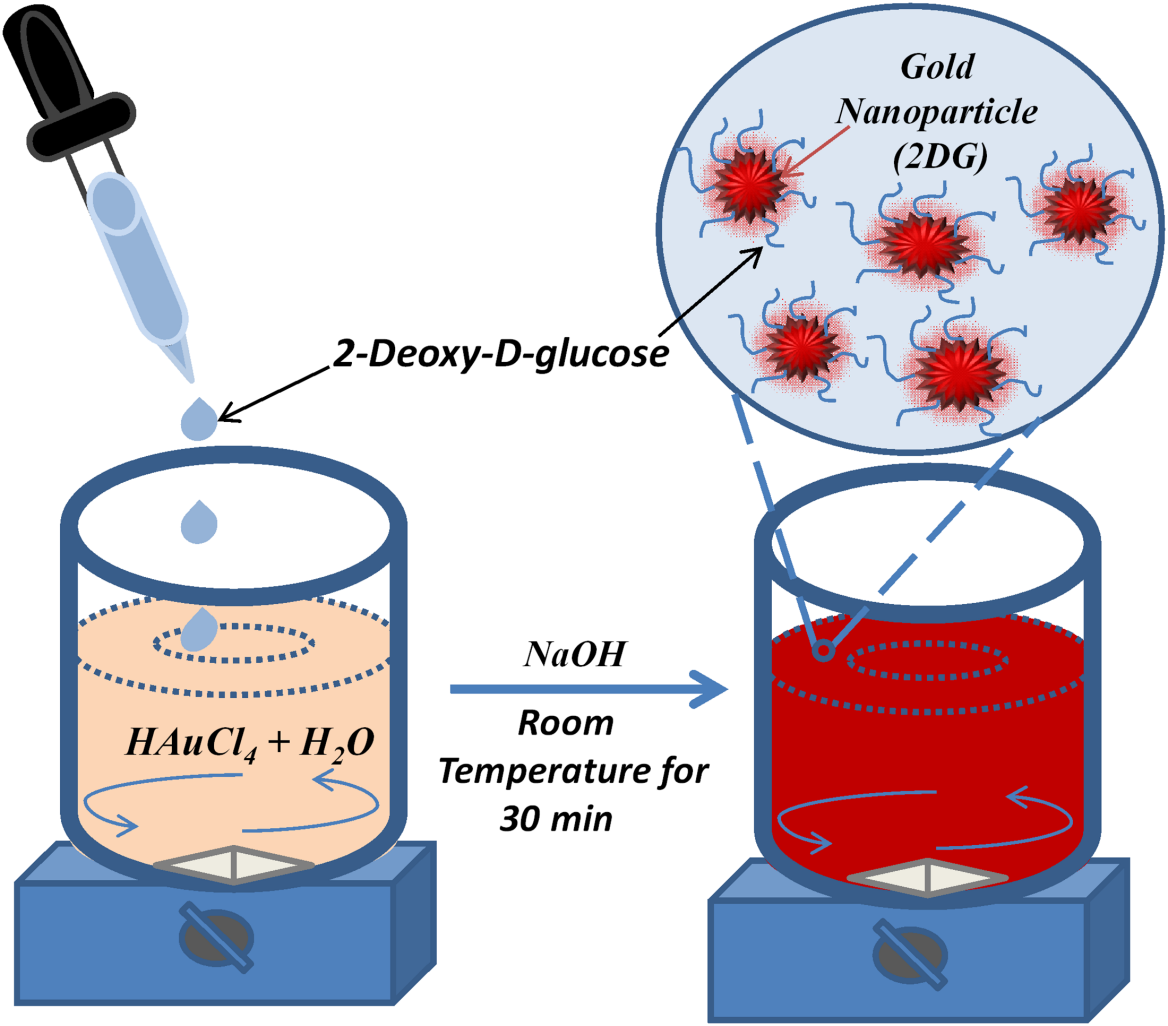
Flow chart of 2DG AuNP. Flow chart of synthesis of 2DG gold nanoparticle at room temperature method.

Synthesis of 2-deoxy-D-glucose-AuNps: Synthesis of 2-deoxy-D-glucose capped gold nanoparticle has been done in our lab for the first time by a half an hour reaction at room temperature. The method briefly described as follows. We synthesized 2-deoxy-D-glucose capped gold nanoparticles by chemical route using HAuCl_4_ and 2-deoxy-D-Glucose. The aqueous solution of 0.05 M HAucl_4._3H_2_O has been added to 2-deoxy-D-glucose (0.04 M) and stirred for 30 minutes. Subsequently, 0.5 M sodium hydroxide (NaoH) is added for completing reduction of gold salt. This resulted in a brisk red colored solution of 2DG-AuNps. 2-deoxy-D-glucose acted as reducing as well as capping agent in the AuNp synthesis as shown in Fig 1.

**Fig 2:**
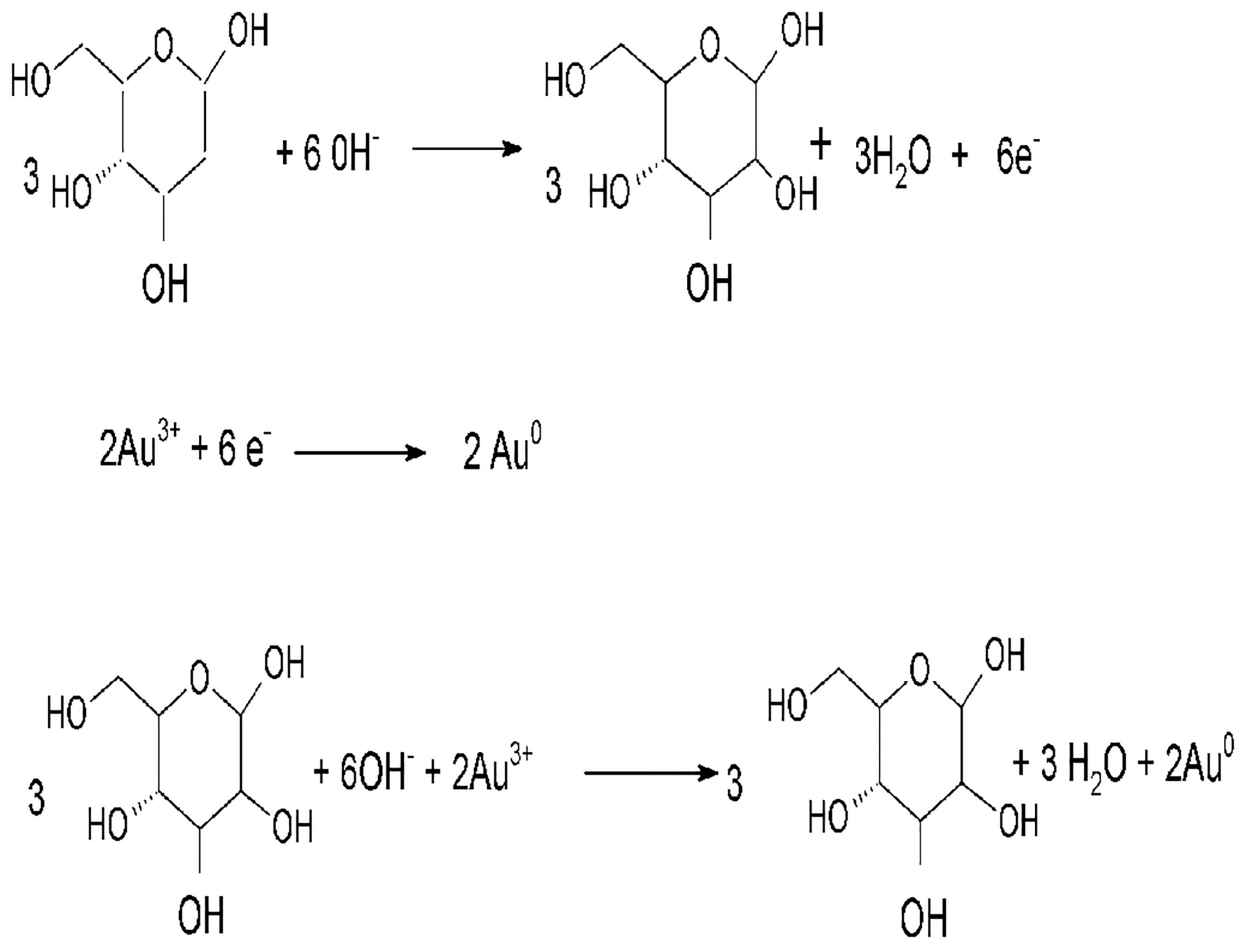
Chemical equation. The reduction reaction equation for the formation of Au nanoparticles.

Capping has been confirmed using FTIR analysis. Chemical equation for the reaction is shown in Fig 2.

Synthesis of Citrate-AuNPs: Citrate- gold nanoparticles have been synthesized using chemical method. Conventional techniques for aqueous synthesis of gold nanoparticles involve reduction of Au(III)Cl_3_ with trisodium citrate [43].

#### Characterization techniques

Gold nanoparticles stabilized with citrate, glucose and 2-deoxy-D-glucose are characterized by UV-Visible absorption spectroscopy (Shimadzu UV-1800). Confirmation of capping of the NPs has been done by FTIR (Shimadzu IR Prestige-21) analysis. Morphology and dimensions of the AuNps are studied through Dynamic light scattering (DLS; Malvern-Instrument) and Transmission electron microscopy (TEM; JEOL 2010 HRTEM). SEM-EDS (Carl Zeiss–ΣIGMA–Oxford Instrument) analysis is performed to verify the presence of gold content in AuNps. SERS study has been done using a micro-Raman setup (RAM HR, Jobin Yvon.).

#### HepG2, Hela cells and HCT 116 treatment with different capping agents of gold nanoparticles

Different cancer cell lines such as HepG2, HeLa and HCT 116 are treated with Glucose-AuNp, 2-deoxy-D-Glucose-AuNps and Citrate-AuNps at concentration ranging from 5 μM to 90 μM per ml of culture medium. The time of incubation of the cells with AuNps was 72 hrs. Throughout the experiments autoclaved AuNps without any centrifugation have been used. After 72 hrs the medium with AuNps was discarded and cells were washed thrice with phosphate buffer saline (PBS) to remove free Glu-AuNps, 2DG-AuNps and Citrate-AuNps.

#### MTT assay

To study the cell viability, MTT assay has been carried out for all the three different cell lines treated with the three differently capped AuNps viz. Gu-AuNps, 2DG-AuNps and Citrate-AuNps. Briefly Hep G2, HeLa and HCT 116 Cells are seeded in 96 well plate at a density of 10^4^ and 10^6^ cells per well prior to treatment with Glu-AuNps, 2DG-AuNps and Citrate-AuNps at various concentrations of the range 5 μM, 10 μM, 20 μM, 30 μM, 50 μM, 70 μM and 90 μM. The Glu-AuNp, 2DG-AuNps and Citrate-AuNPs solution are used as positive control. 10 μl of MTT reagent (stock solution-5 mg/ml in PBS) is added in each well and incubated for 2.5 hrs in incubator (37°C, 5% CO_2_ and 95% humidity). After careful removal of the medium from the well, 100 μl of DMSO is added to each well followed by measuring the absorbance of Formazan at 595 nm by a BIO-RAD iMark^™^ micro plate reader. MTT assay has been performed in triplicate, Error bars are SEM (n = 8) for MTT assay one-way ANOVA has been done and p<0.05 has been considered as the level of significance.

## Results and discussion

### UV-Visible spectroscopy of gold nanoparticles

**Fig 3:**
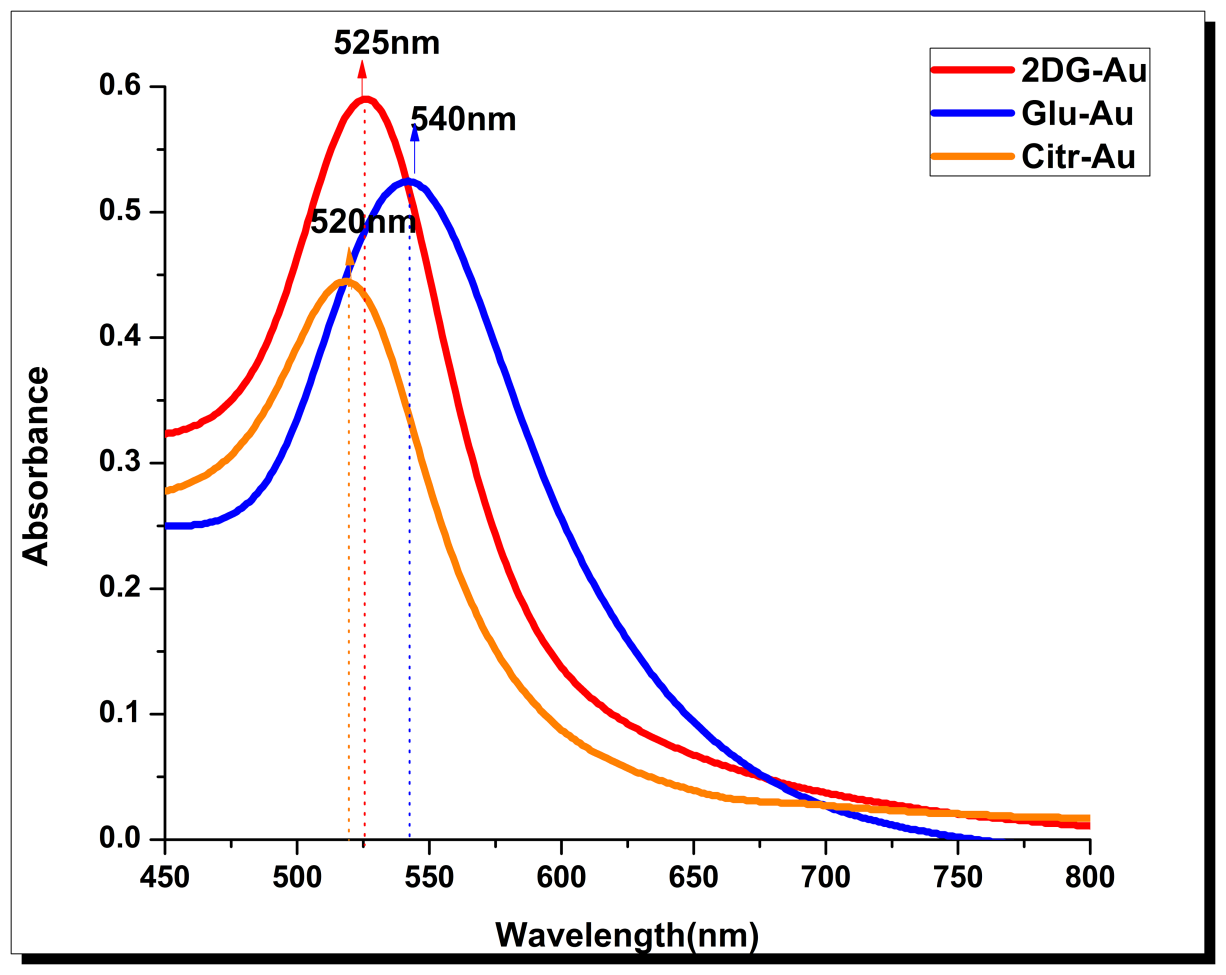
UV-visible absorption spectrum of AuNPs. **A** UV-visible absorption spectrum of glucose-AuNps showing SPR peak at 540 nm; **B** Citrate-AuNps showing SPR peak at 520 nm; **C** 2DG-AuNps showing SPR peak at 525 nm.

Fig 3 represents the absorption spectrum of the three differently capped AuNps. The strong absorbance peaks observed at 540 nm, 525 nm and 520 nm for Glu-AuNps, 2DG-AuNps and Citrate-AuNps respectively, show the presence AuNps.

The absorbance maxima of the three respective AuNps are confirmed from earlier reports [42,43]. During citrate-AuNp synthesis, citrate acts as reducing (Au^3+^ to Au^0^) as well as capping agent. In the case of Glu-AuNp synthesis, glucose acts as reducing (Au^3+^ to Au^0^) as well as capping agents in alkaline aqueous environment due to addition of NaOH. Glucose is bound to surface of nanogold by hydrogen bonding of hydroxyl (-OH) group and similar changes observed in case of 2-deoxy-D-Glucose.

### FT-IR analysis of gold nanoparticles

**Fig 4:**
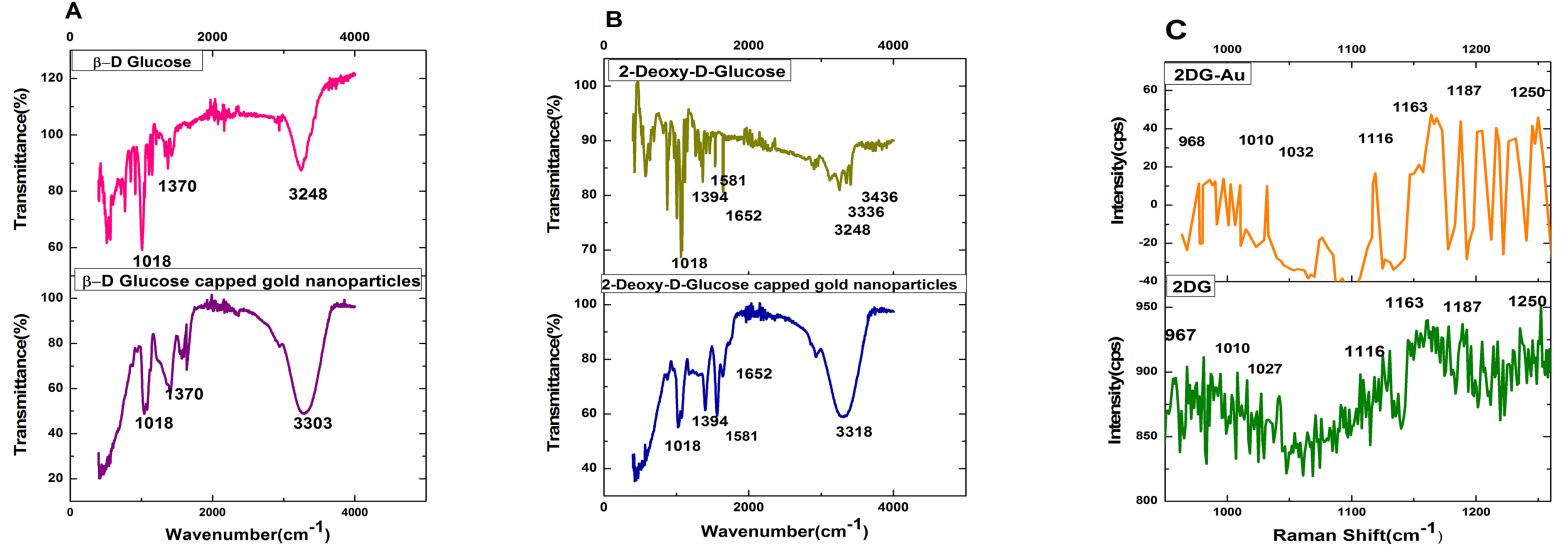
FTIR and SERS spectrum analysis. FTIR spectrum of (**A)** glucose and glucose-gold nanoparticles; (**B)** 2DG and 2DG-gold nanoparticles; **C**: SERS Raman spectra of 2DG-Au and Raman spectra of 2DG.

Characterization of the particles using FT-IR shows the interaction between gold nanoparticles and the reducing agents (Fig 4).

FT-IR spectrum shows strong absorption peak at 3303 cm^-1^ (Fig 4A), manifesting the presence of β-D-glucose as an essential component of the Glu-AuNp. In addition, the absorption band of the –OH stretching undergoes a significant high frequency shift observed at 3303 cm^-1^ suggesting the intimate association between β-D-glucose and the surface of the Au nanoparticles [42]. FT-IR analysis of 2-deoxy-D-Glucose-AuNp exhibited a characteristic peak at 3318 cm^-1^ attributed to the stretching vibrations of -OH, which is assigned to –OH absorbed by gold nanoparticles (Fig 4B). The presence of characteristic bands in 1600–1000 cm^-1^ region corresponds to skeleton vibration of 2DG molecules. The hydrogen bond of chelate type appears as a wide degraded band in region 3500–3200 cm^-1^. The intensive bands in 1394–1018 cm^-1^ region observed in 2-deoxy-D-Glucose-AuNp correspond to deformation vibration of hydroxyl group. Well-defined peaks in the fingerprint region between 1000–1600 cm^-1^ confirms 2DG molecule adsorption on gold surface as these peaks corresponds to 2DG as established by earlier workers [44].

### TEM analysis of gold nanoparticles

**Fig 5:**
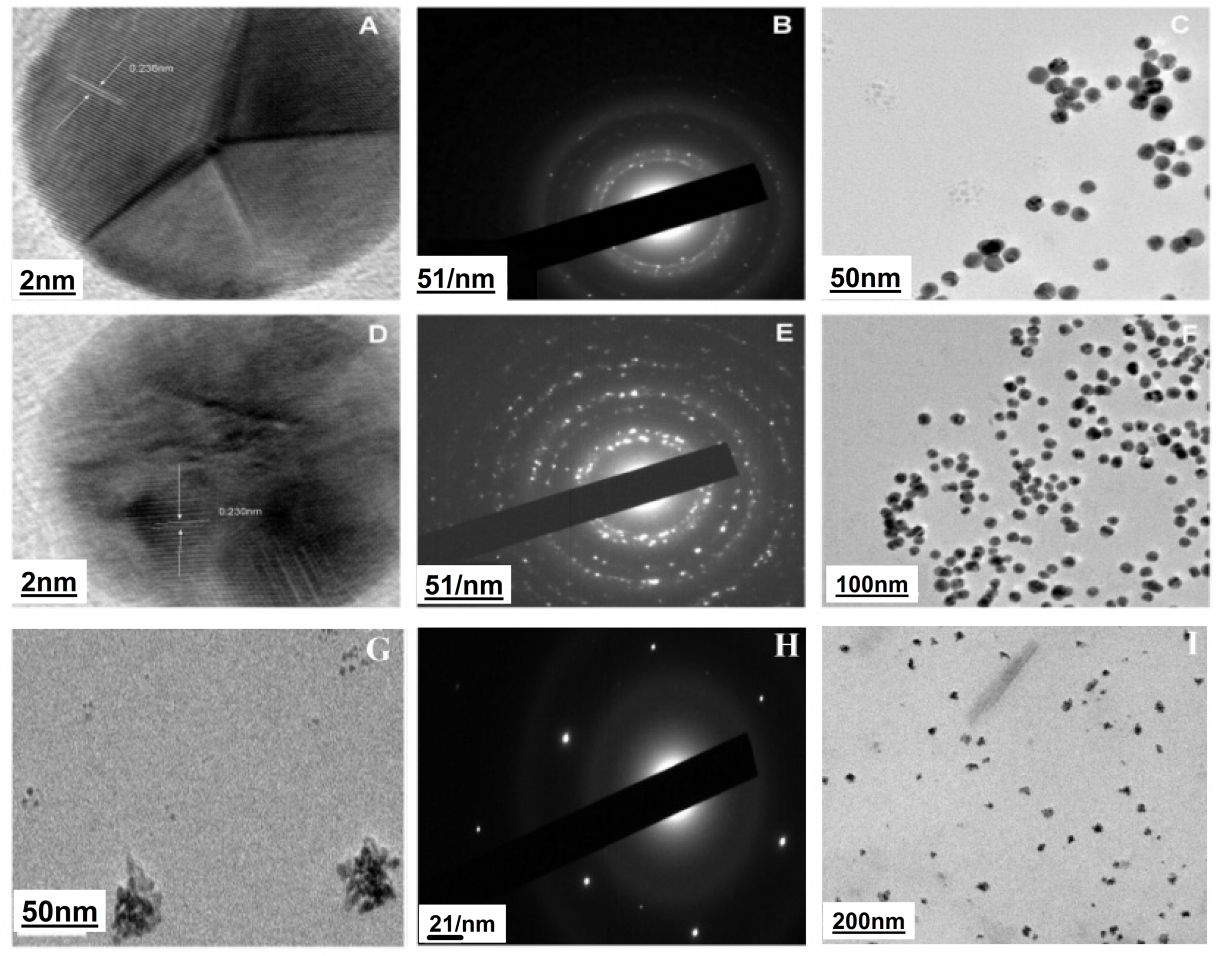
TEM analysis of AuNPs. **5A, 5C** High magnification TEM image of glucose capped gold nanoparticles showing FFT measurement using Image-J software; **5B** SAED pattern of glucose capped gold nanoparticles; **5D, 5F** High magnification TEM image of citrate -gold nanoparticles showing FFT measurement using Image J-software; **5E** SAED pattern of citrate—gold nanoparticles; **5 G, 5I** Shows the high magnification TEM image of 2-Deoxy-D-Glucose capped gold nanoparticles;5 **H** SAED pattern of 2DG-Capped gold nanoparticles.

The size and shape of the gold nanoparticles using different capping agents as observed by TEM is shown in Fig 5.

**Fig 6:**
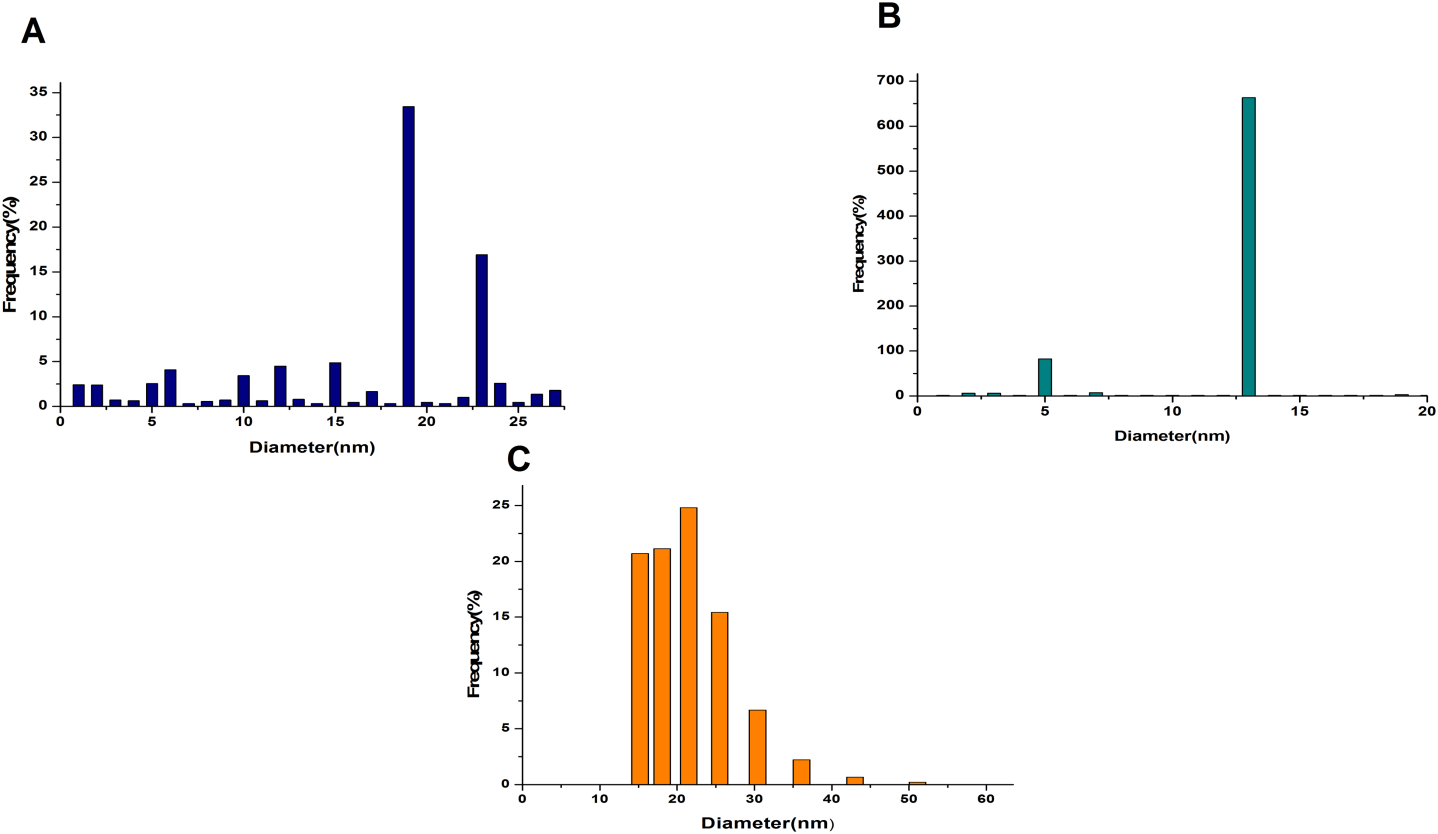
Size distribution measurement plot of AuNPs. Size distribution measurement curve of (**A)** glucose-gold nanoparticles; (**B)** citrate-gold nanoparticles; **C** Dynamic light scattering measurement of 2DG coated gold of size 21.51nm.

**Fig 7:**
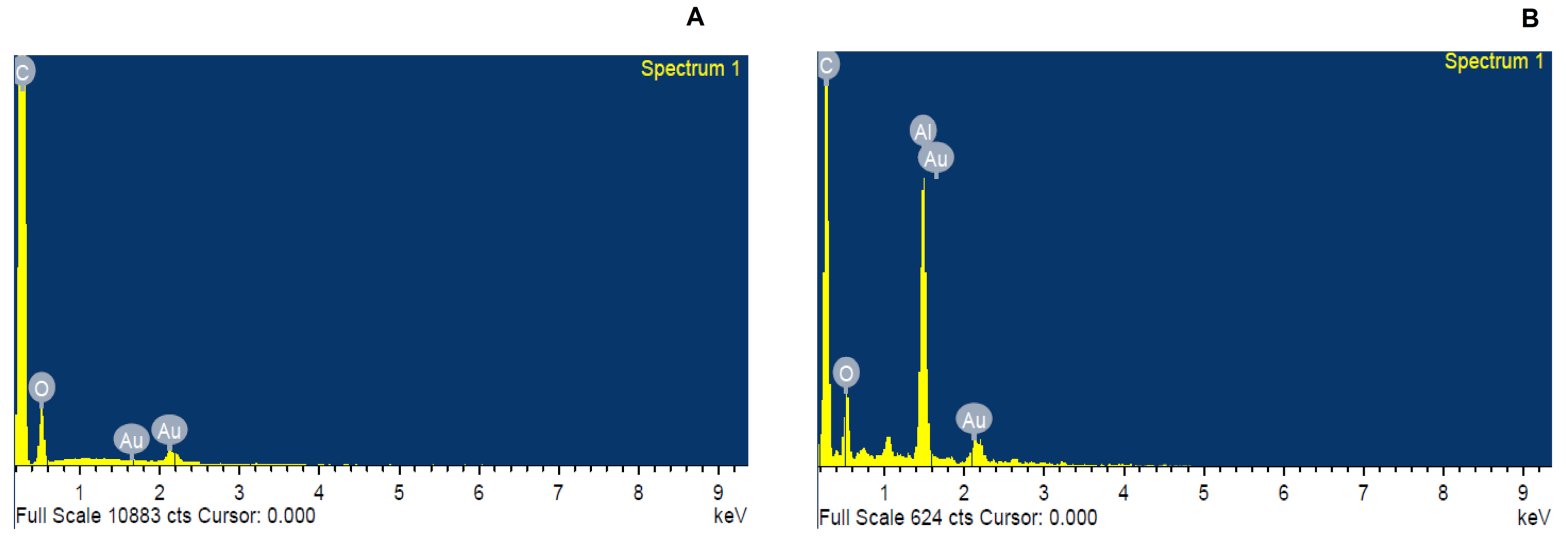
SEM-EDS spectrum of glucose-capped gold nanoparticles and 2DG-capped gold nanoparticles. SEM-EDS analysis of (**A)** glucose-capped gold nanoparticles; (**B)** 2DG-capped gold nanoparticles;

TEM analysis show triangular shape of the glucose capped gold nanoparticles (Fig 5A and 5C) with narrow particle size-distribution of range 19.05±1.13 nm and sigma = 0.15. Furthermore, a few spherical gold nanoparticles also appeared in the image. ED pattern of these gold nanoparticles corresponded well to the crystalline planes of the face-centered-cubic (fcc) structured gold (Fig 5B), suggesting the crystalline nature of these Au nanoparticles. In addition, the multiple lattice fringes with an interplanar spacing of 2.36 A^0^ consistent with the interplanar distance of (111) plane can be observed clearly by using high-resolution TEM which also confirms the crystalline nature of the Glu-Au nanoparticles. In contrast to the triangular shape of the particles in Glu-AuNps, the citrate capped gold nanoparticles show spherical shape (Fig 5D and 5F) with majority of the particles having similar size of 14.87 nm (sigma = 0.15) and inter planar spacing of 2.36 A^0^. TEM image of citrate capped gold nanoparticles indicates a relatively high monodispersity of the Au nanoparticles formed in the system as compared to Glu-AuNps which confirms earlier reports [45]. Fig 5G–5I represent the TEM images of the 2DG-AuNps where ED pattern of these nanoparticles is shown in Fig 5H. Interestingly the 2DG-AuNps show urchin shape of the gold nanoparticles under TEM with a diameter of 21.51 nm, as observed by particle size analysis using DLS. Zeta potential value of 2DG-AuNp is -19.1 mv and has been observed to be stable for 8 months. The particle size distributions in the three differently capped AuNPs are shown in Fig 6,

Histogram plot in the Fig 6A represents the size distribution of Glu-AuNp, size distribution of the citrate-Au-nanoparticles is presented in the Fig 6B and 6C represents the size distribution of 2DG-AuNp, Presence of gold in the nanoparticles (Glu-AuNps and 2DG-AuNps) is confirmed by SEM-EDS analysis (Fig 7).

### SERS analysis of gold nanoparticles

Surface-enhanced Raman scattering/spectroscopy (SERS), a vibrational spectroscopic method that provides information about molecular position, has raised a great interest as a sensitive technique for chemical and bioanalytical sensing and imaging [46–48]. Different shaped nanoparticle envisioned that these particles can be utilized to catalyse many other reactions, assembled to form super lattice structures, and serve as excellent SERS substrates [49]. When the molecules are adsorbed onto metallic surfaces with nanometer-scale roughness or onto metal nanoparticles, an enhancement in intensity of Raman scattering is observed[50,51] Utilising this, there exists a plethora of work which has been performed to demonstrate SERS effects for different molecules, especially with different shaped nanoparticles [52,53]. Properties of the gold nanoparticles are dependent on their sizes, shapes, and crystallity [54]. Gold nanoparticles have been used as effective surface-enhanced Raman spectroscopy (SERS) substrates for decades [55]. Our present study shows good SERS property of the 2DG-AuNp with 50 times increase in Raman signals (Fig 3C). The peak at 1032 cm^-1^ corresponds to C-H in plane bending and the Raman signal at 1163 cm^-1^ might appear due to C-H out of plane bending as a strong signal. Our observation finds support from earlier report [56]. At 967 cm^-1^ medium Raman signal observed is possibly due to C-O-C bond, while peak at 1116 cm^-1^ may be associated with orientation of C-O groups. Reports from earlier researchers also showed some bands at _˜_ 1263 cm^-1^ assigned at complex modes of CH_2_OH groups [57]. It is known well that in contrast to semiconductor or insulator nanomaterials, the optical properties of metal nanoparticles mainly depend on size and shape of the particles where, more sensitivity towards shape and less so to size is seen [58]. Recently it has been shown that the SERS enhancement factor generated from the gold nanoparticles increases as the size of nanoparticles increases [59]. However, the exact mechanism of SERS is still not clearly understood. Several groups have been working to explain the origin of SERS, and currently, the amplified electromagnetic (EM) field at the surface of the metal substrate is being considered as the main source of enhancement [60]. Recent studies have reported metal film over nanosphere surfaces as excellent candidates for experiments that were once impossible with more primitive SERS substrates [61]. Additionally, the colloidal nanostars showed the highest SERS enhancement factor while the nanospheres possessed the lowest SERS activity under different excitations[62]. Plasmonic gold nanostars offer a new platform for surface-enhanced-Raman-scattering (SERS). However, in conditions with the presence of organic surfactant on the nanoparticles, SERS characterization and application of nanostar in solution is a challenging factor in the field of SERS bioapplications [63]. Interestingly, FT-SERS studies showed that branched gold particles had stronger SERS activity relative to the nanobranched ones, which made these particles very attractive in the SERS applications [64]. In recent years, developments and applications of surface-enhanced Raman scattering(SERS) nanosensors and nanoreporters in biochemical monitoring, medical diagnostics, and therapy have been reported [65]. As gold nanoparticles that exhibit strong SERS properties are considered to be utilized for the development of biosensors and biocatalyst, the urchin like shaped gold nanoparticles (2DG-AuNps) synthesized by our group may be considered as more potent for biomedical applications because of their tunable SPR properties and excellent SERS enhancement ability. It has been shown earlier that such shaped nanoparticles are better candidates for applications in the optical imaging-based disease theranostics[66].

### Effect of nanoparticles on the cell viability

**Fig 8:**
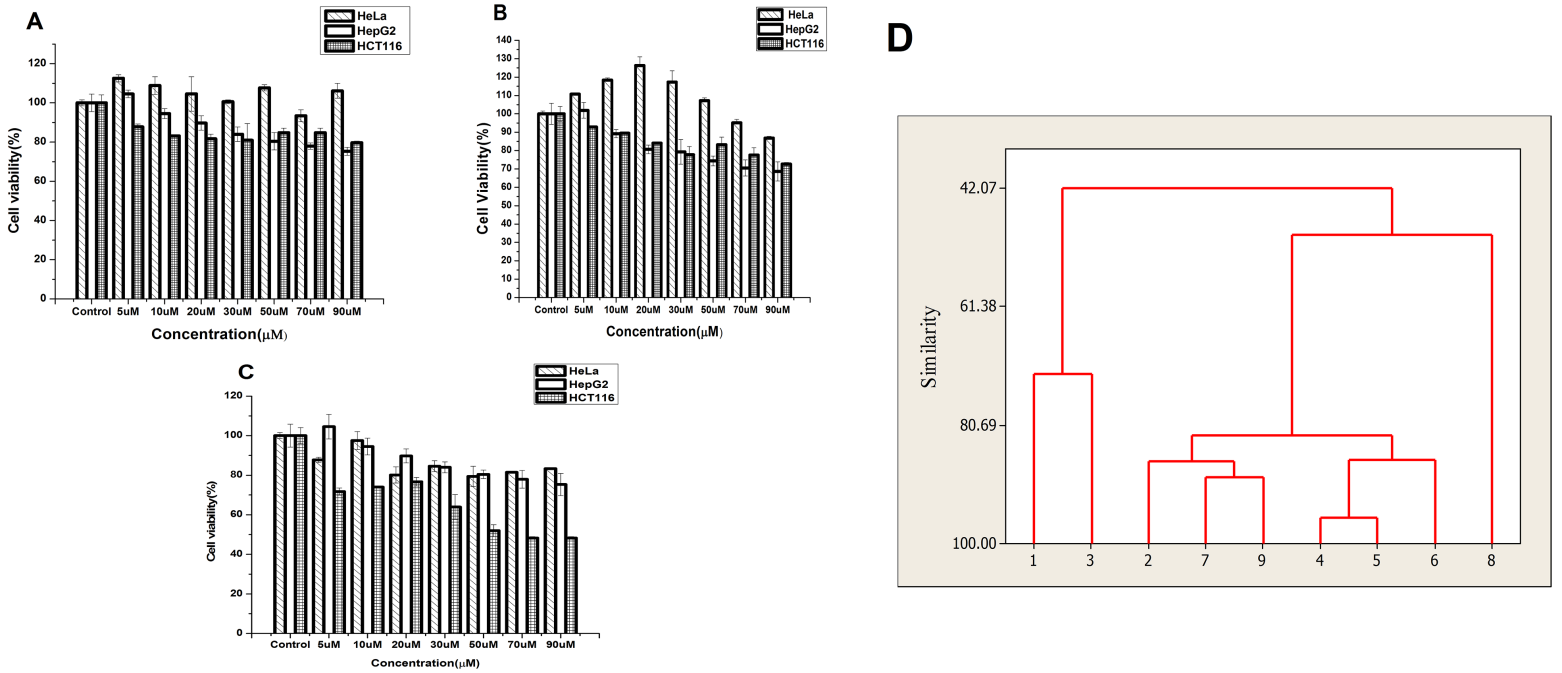
Cell viability plot and dendrogram plot. **A** Viability of HeLa cells, HepG2 cells and HCT 116 cells treated with 2-Deoxy-D-Glucose-gold nanoparticles; **B** Viability of HeLa cell line; HepG2 cell line and HCT116 cells treated with glucose capped gold nanoparticles; **C** Viability of HeLa cells, HepG2 cells and HCT 116 cells treated with citrate-gold nanoparticles. Standard deviation shown as error bar, p<0.05. **D**: Dendrogram shows cell lines are aligned separately and grouped based on similarities in their cytotoxicity expression using a hierarchical clustering analysis technique. 1 represents 2DG-AuNp-Hela,2: 2DG-AuNp-HepG2, 3:2DG-AuNp-HCT116, 4:Glu-AuNp-Hela,5:Glu-AuNp-HepG2,6: Glu-AuNp-HCT116,7: Citr-AuNp-Hela,8: Citr-AuNp-HepG2, 9: Citr-AuNp-HCT116.

Comparative analysis of the MTT assay data indicates that the three differently capped AuNps impart differential effects on survival of the three different types of cell lines (Fig 8A, 8B and 8C).

2DG-AuNp show negligible cell death in HCT 116 cells with no significant physiological state change of the cells, and manifested a survival of ≥ 80± 0.70% in all the concentration ranging from 5–90 μM (Fig 8A). Results show that these nanoparticles are efficiently taken up by the cells and possess no cytotoxicity. Similar to this, Glu-AuNps also imparted no toxicity in HCT 116 cells and a 73 ± 0.84% cell viability has been observed in case of cells treated with 90 μM (Fig 8B). However, in case of HCT 116 cells treated with citrate –AuNps a drastic drop of cell survival has been observed in cells treated with higher concentrations (50, 70, 90 μM) of the particles (Fig 8C). The present data indicating that higher concentration of citrate capped gold nanoparticles are toxic to HCT 116 cells, is in perfect harmony with the findings of some earlier researchers who reported similar observations and postulated that such toxicity might be associated with the acidic nature of citrate, the capping agent of the concerned nanoparticles [67]. Toxicity imparted by citrate –AuNp may also be due to the spherical shape of the particles supporting some earlier study who reported higher toxicity of gold nanospheres when compared to that of gold nanostars [68]. Interestingly, the present results reflect the potential of two of the synthesized nanoparticles to stimulate cell growth. Such stimulation of cell growth, however has been observed not only to be restricted to particular cell type but also depends upon the capping and shape of the nanoparticles. 5–50 μM concentrations of both Glu-AuNps and 2DG-AuNps have been observed to boost survival in Hela cells. In contrast to 90 μM Glu-AuNps showing 87±0.84% viability of Hela cells (Fig 8B), same concentration of 2DG-AuNps manifested an increase in cell survival (Fig 8A). 2DG-AuNp-induced proliferative response of Hela cells has been reported by earlier researchers [69]. Citrate –AuNps showed no boosting in Hela cells but maintained ≥ 80± 0.84% viability of the cells when treated in the same concentration range (Fig 8C). HepG2 cells showed similar boost in cell growth when treated with 5 μM of all the three types of AuNps.

Fig 8D shows dendrogram of survivability of all the cell lines treated with the differently capped gold nanoparticles. Hierarchical clustering analysis manifest high degree of similarity between the 2DG-AuNp and Glu-AuNp treated Hela cells. While in case of HCT116 the differentially capped glucose AuNps also form a cluster (with similarity > 80.69), maximum similarity is observed between 2DG –Au Np and citrate- AuNps. In contrast, citrate-AuNp-HCT116 showing cytotoxicity is separated out from the clusters. Thus, results of the present investigation show among the three types of AuNps, while Glu-AuNps and 2DG-AuNps possess no cytotoxicity, 2DG-AuNp is most effective in maintaining survival in the three types of cancer cells especially Hela cells. 2-deoxy-D- glucose has been reported earlier to be efficient in targeting tumor cells [70], which could be a perfect candidate for coupling to Au-nanoparticles to have biomedical applications, especially to target glucose-dependent cancer cells sparing the normal tissues and deliver the coupled drug to the site of interest[71,72]. Our present study portrays the possibility of 2-deoxy-D-glucose capped gold nanoparticles as a better candidate for theranostic application.
